# Diving into Health: A Mixed Methods Study on the Impact of Scuba Diving in People with Physical Impairments

**DOI:** 10.3390/healthcare11070984

**Published:** 2023-03-30

**Authors:** Tania Santiago Perez, Brandi M. Crowe, Patrick J. Rosopa, Jasmine N. Townsend, Michael R. Kaufman

**Affiliations:** 1 Recreational Therapy, Department of Counseling, Recreation & School Psychology, Florida International University, 11200 SW 8th St ZEB 336-A, Miami, FL 33199, USA; 2 Recreational Therapy, Department of Parks, Recreation & Tourism Management, Clemson University, 105 Sirrine Hall, Clemson, SC 29631, USA; 3 Department of Psychology, Clemson University, 410J Bracket Hall, Clemson, SC 29631, USA; 4 Therapeutic Scuba Institute, 4001 SW 132nd Avenue, Miramar, FL 33027, USA

**Keywords:** adapted/adaptive scuba diving, physical impairment/disability, social identity, physical health, social health, psychological health, self-efficacy, health-related quality of life, disability level, rehabilitation in disability

## Abstract

The impacts of scuba diving on people with physical impairments are unknown. Grounded on the social identity approach to health, the aim of this study was to test and describe the relationships between scuba diving social identity, self-efficacy, social health, psychological health, physical health, health-related quality of life (HRQOL), and disability level among recreational scuba divers with physical impairments. A mixed methods explanatory sequential design was employed. The quantitative strand used an 80-item cross-sectional survey, with the data analyzed via a path analysis. The qualitative strand used 1:1 interviews across 3 case study groups; the data were analyzed using deductive and inductive analyses. Mixing occurred via a joint display with meta-inferences. The quantitative results (*n* = 78) indicated that self-efficacy was a significant predictor of social health, psychological health, physical health, HRQOL, and disability level. The qualitative findings (*n* = 15) consisted of six themes, whereby participants described scuba as a positive social identity that provides them with meaning, purpose, and belonging. Furthermore, they described scuba diving as a positive contributor to their self-efficacy, social health, psychological health, physical health, and quality of life. During the mixing of data, the quantitative and qualitative results did not match on the influence of scuba diving social identity on self-efficacy, social health, psychological health, physical health, HRQOL, and disability level. A further analysis revealed that the range restriction impaired the conclusive quantitative evidence on the scuba diving social identity variable. The meta-inferences derived from the data integration suggest that scuba diving plays a role in the self-efficacy, health, HRQOL, and disability level among scuba divers with physical impairments. The findings point to the potential of scuba diving as a health promotion recreational activity and rehabilitation modality for people with physical impairments.

## 1. Introduction

Physical or mobility impairments caused by musculoskeletal and neurological conditions account for nearly two-thirds (65.6%) of the total physical rehabilitation needs worldwide [1]. People with physical impairments experience significant health disparities [2,3] and lower levels of health-related quality of life (HRQOL) [4,5,6] compared to people without disabilities. The health disparities experienced by people with physical impairments encompass their physical, psychological, and social health. Physically, people with physical impairments experience more chronic pain and difficulties performing many activities of daily living [7]. Psychologically, they experience higher levels of anxiety and depression [7]. Socially, they experience more isolation, less social support, and less social participation [7]. Thus, innovative approaches that promote positive health and HRQOL among people with physical impairments are needed. 

An emergent approach in health psychology, called the social identity approach to health [8,9,10], is an alternative to the current biomedical, psychological, and social capital approaches to health. This approach reverses Engel’s biopsychosocial [11] model of health into a sociopsychobio model [12] by putting the social domain of health at the forefront of the analysis of health. The social identity approach to health explains that group memberships and social identities have consequences for people’s health; a person’s social identity either decreases, sustains, or increases their health [9,10,12,13,14,15]. This approach, derived from social identity [16] and self-categorization [17] theories, encompasses two fundamental propositions. First, it proposes that participation in social groups is a major determinant of health. Second, it proposes that the impact a particular social group has on an individual’s health depends on the nature of the social identity that underpins that particular group membership [9]. These two propositions suggest that social groups and their derived social identities can have a significant impact on the health and HRQOL of individuals [8,9,10]. The social identity approach to health posits that health is enhanced through identification with social groups that provide social and psychological resources such as connection, meaning, support, efficacy, and control [8,9,10]. Recreation groups may provide those psychological resources to individuals with physical impairments and enhance their health. 

Due to being self-determined, meaningful, and enjoyable endeavors [18], recreation and leisure activities can provide some of the richest contexts for the development of positive social identities and opportunities for experiencing competence and self-efficacy [19]. Recreation groups allow individuals to be part of a group of people who have similar interests, skills, and needs, aiding in their development of a sense of belonging. Additionally, recreation groups provide individuals with important social connections, feedback, and social support. Recreation and leisure groups can build social identity through a community of acceptance, affective involvement, symbols of identification, and group meaning [20]. Despite the growing evidence supporting the social identity approach to health and the benefits of social group memberships, little attention has been given to recreation social identities as important predictors of health outcomes. One nearly unexplored recreational social identity that may be related to higher levels of health, HRQOL, and self-efficacy among people with physical disabilities is being a scuba diver. 

Scuba diving is a recreational activity that provides an opportunity to experience a novel, meaningful, and positive social identity. Becoming a scuba diver allows individuals to belong to a community with shared skills, needs, and interests, which may promote social relationships, friendships, and feelings of belonging. The evidence on the health benefits of scuba diving among people with physical impairments is scarce [21]. While limited, the available literature suggests that being a scuba diver might promote higher levels of health among scuba divers with physical impairments [22,23,24,25,26,27,28,29,30,31,32,33,34]. Socially, scuba diving has been reported to increase the ability of individuals to engage in social interactions [22], as it provides opportunities for social comfort [35]. Scuba may be an important contributor to the quality of life of divers with physical impairments through providing enhanced social circles [32], unique social experiences [32], and an improved perception of social support and psychosocial status [23]. Additionally, scuba exposes individuals to a natural environment, which can have calming and stress-reducing effects [30]. Within the components of scuba diving there are similarities with meditation and mindfulness techniques, as scuba favors a state of full consciousness and openness associated with slow and ample breathing [29]. Some psychological outcomes reported by scuba divers with physical impairments include increased state-level mindfulness and contentment [27], improvements in the symptomology of anxiety and depression [28], enhanced self-esteem and self-confidence [22], experiencing a sense of freedom [33,34], and improved self-concept [23,24,32]. The results of studies on physical health outcomes in people with physical impairment suggest that scuba may reduce the spasticity and frequency of muscle spasms [25,26], decrease chronic pain [22,28], increase pulmonary vital capacity [26], improve circulation and body strength [34], and increase general physical ability [22,31]. The current available evidence suggests a relationship between being a scuba diver and health outcomes. Thus, this study addressed the following research questions. 

### Research Questions and Hypotheses

Mixed methods question: To what extent does scuba diving social identity relate to self-efficacy, social health, psychological health, physical health, HRQOL, and disability level among people with physical impairments? (QUAN → QUAL)

Quantitative research question (QUAN): What are the relationships between scuba diving social identity and self-efficacy, social health, psychological health, physical health, HRQOL, and disability level among people with physical impairments?

**Hypothesis** **1.** *There is a positive causal relationship between scuba diving social identity and self-efficacy, social health, psychological health, physical health, and HRQOL among people with physical impairments.*

**Hypothesis** **2.** *There is a negative causal relationship between scuba diving social identity and disability level among people with physical impairments.*

**Hypothesis** **3.** *Self-efficacy mediates the relationship between scuba diving social identity and social health, psychological health, physical health, HRQOL, and disability level among scuba divers with physical impairments.*

Qualitative research question (QUAL): How do scuba divers with physical impairments explain their scuba diving social identity in relation to their self-efficacy, social health, psychological health, physical health, HRQOL, and disability level?

To date, there appear to be no publications on the relationship between scuba diving social identity and health grounded on the social identity approach to health. Thus, the proposed study intends to advance the understanding of the social identity approach to health by exploring scuba diving social identity as a health-enhancing identity among people with physical impairments. Specifically, this study aims to test and describe the relationships between scuba diving social identity and levels of self-efficacy, social health, psychological health, physical health, HRQOL, and disability among people with physical impairments. 

## 2. Materials and Methods

### 2.1. Design

**Figure 1:**
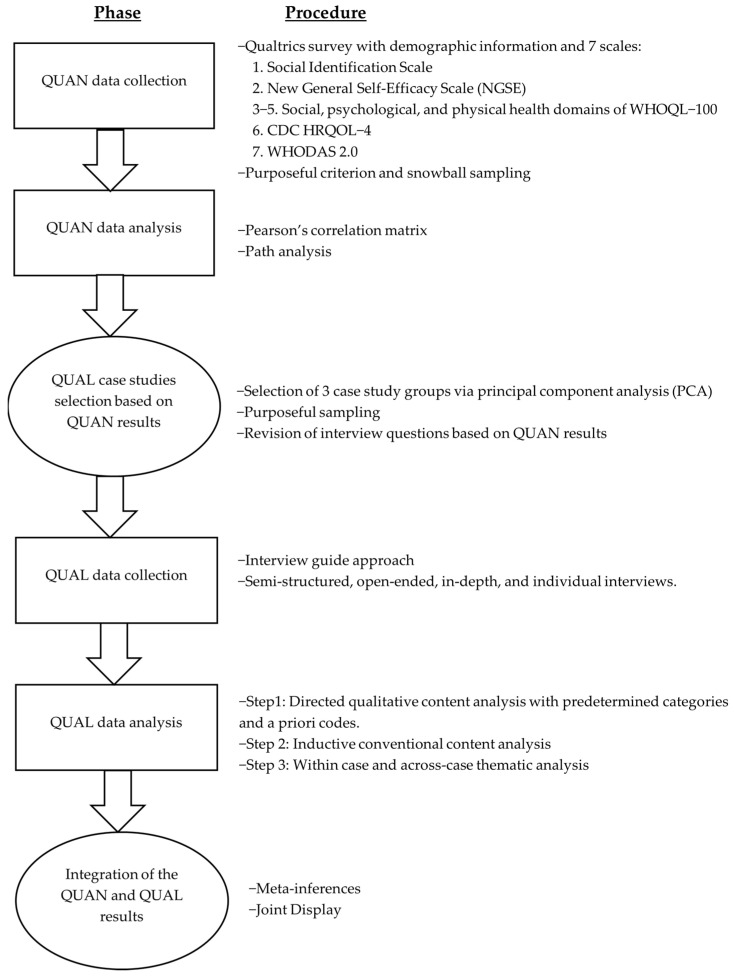
Mixed methods design diagram.

This explanatory sequential mixed methods design [36] commenced with a quantitative phase, followed by a qualitative phase. During the quantitative phase, a cross-sectional survey was conducted to quantify and explore through path analysis the relationships between 7 variables: (1) scuba diving social identity, (2) self-efficacy, (3) social health, (4) psychological health, (5) physical health, (6) HRQOL, and (7) disability level. Using a multiple case study approach [37], the follow-up qualitative phase involved 1:1 semi-structured interviews with 3 case study groups representative of participants who were high, average, or low scorers on the dependent variables assessed through the quantitative survey. The qualitative phase aimed to explain quantitative findings and add depth to the investigation of the relationship between the variables from the participants’ views. Figure 1 displays the study’s mixed methods procedure. 

### 2.2. Sampling and Recruitment

Inclusion criteria for the study entailed individuals who: (a) were aged 18+; (b) self-reported having a physical impairment; and (c) self-identified as a recreational scuba diver. The exclusion criteria involved individuals who self-identified: (a) as a scientific, professional, technical, military, or commercial diver; (b) as having an intellectual disability. Using purposeful criterion [38] and snowball sampling [39] techniques, participants were recruited for the first quantitative phase. Organizations globally that promote and train people in adapted scuba diving were contacted via phone, email, and social media, and provided with recruitment flyers to distribute among their members. The target sample size (n) for the quantitative phase of the study was determined with a priori power analysis using G*Power 3.1 [40] statistical software. Assuming a multiple linear regression, the parameters that were specified were α = 0.05, three predictors in the model at once, and a power of at least 0.8 (1 − β = 0.8). For the effect size, a range from medium to large effect size (*f* ^2^ = 0.15 to 0.3) [40] was used, yielding a target sample size of 41 to 77 participants. A principal component analysis [41] was used to categorize participants from the quantitative phase into 3 case study groups for the qualitative phase: (1) low, (2) average, and (3) high scorers across the 5 dependent variables of social health, psychological health, physical health, HRQOL, and disability level. Five participants per case study group were purposefully selected for the qualitative phase to represent the participants that had the highest, lowest, and average scores among the quantitative sample. The participants shared their experiences from their real-life context as recreational scuba divers with physical impairments; no participant was in a clinical setting or receiving scuba as a clinical intervention. A qualitative methodology was used to explore and describe the relationships between variables in more depth from the views of the participants. 

Prior to starting the study, ethical approval was obtained from Clemson University’s Institutional Review Board (IRB2021-0760, D-2 category). Participation in the study was voluntary, and informed consent was obtained prior to participation. Research participants were informed of their rights as volunteers in the study, and they consented to the publication of the study’s results. 

### 2.3. Phase One: Quantitative Phase

#### 2.3.1. Quantitative Variables and Data Collection

An online retrospective cross-sectional survey via Qualtrics^®^ XMCore [42] was employed to collect data. The participants were asked to reflect on their past or present scuba diving experiences and answer ex post facto. The data were collected via standardized tools on 7 variables: (1) scuba diving social identity, (2) general self-efficacy, (3) social health, (4) psychological health, (5) physical health, (6) HRQOL, and (7) disability level. Demographic data were also collected, including the physical impairment category, impairment onset, gender, age, race or ethnicity, length of scuba diving group membership, diving level, and number of dives logged per year. 

The 4-item Social Identification Scale [9,43] was employed to measure scuba diving social identity using a seven-point Likert scale, or which the maximum score is seven (high social group identification) and the minimum score is one (low social group identification). Self-efficacy was measured with the New General Self-Efficacy Scale (NGSE) [44], an eight-item scale with items presented on a five-point Likert scale from *strongly disagree* = 1 to *strongly agree* = 5; total scores of 4–5 indicate high general self-efficacy while scores of 1–2 indicate low general self-efficacy. Social health was measured using the social relationships domain of the World Health Organization Quality of Life (WHOQOL-100) instrument [45], which consists of 12 items presented in five-point Likert scales and divided in 3 facets: (1) personal relationships, (2) social support, and (3) sexual activity. Psychological health was measured using the psychological health domain of the WHOQL-100 [45], which consists of 20 items presented in five-point Likert scales and divided in five facets: (1) bodily image and appearance; (2) negative feelings; (3) positive feelings; (4) self-esteem; (5) thinking, learning, memory, and concentration. Physical health was measured using the physical health domain of the WHOQL-100 [45], which consists of 12 items presented in five-point Likert scales and is divided into 3 facets: (1) energy and fatigue, (2) pain and discomfort, and (3) sleep and rest. Social health, psychological health, and physical health scores were transformed linearly from their 5-point ordinal scales to 0–100 scores [46], with higher scores indicating higher levels of health. HRQOL was measured using the Centers for Disease Control and Prevention (CDC) HRQOL-4 instrument [47,48], a core set of four questions on HRQOL producing final scores between 0 (very poor HRQOL) and 4 (very high HRQOL). The disability level was measured using the World Health Organization Disability Assessment Schedule short version (WHODAS 2.0), a 12-item assessment of disability that uses a five-point Likert scale on the difficulty experienced when completing activities from 0 *= none* to 4 = *extreme or cannot do,* producing final scores ranging from 0 (no disability) to 48 (complete disability). 

#### 2.3.2. Quantitative Data Analysis

**Figure 2:**
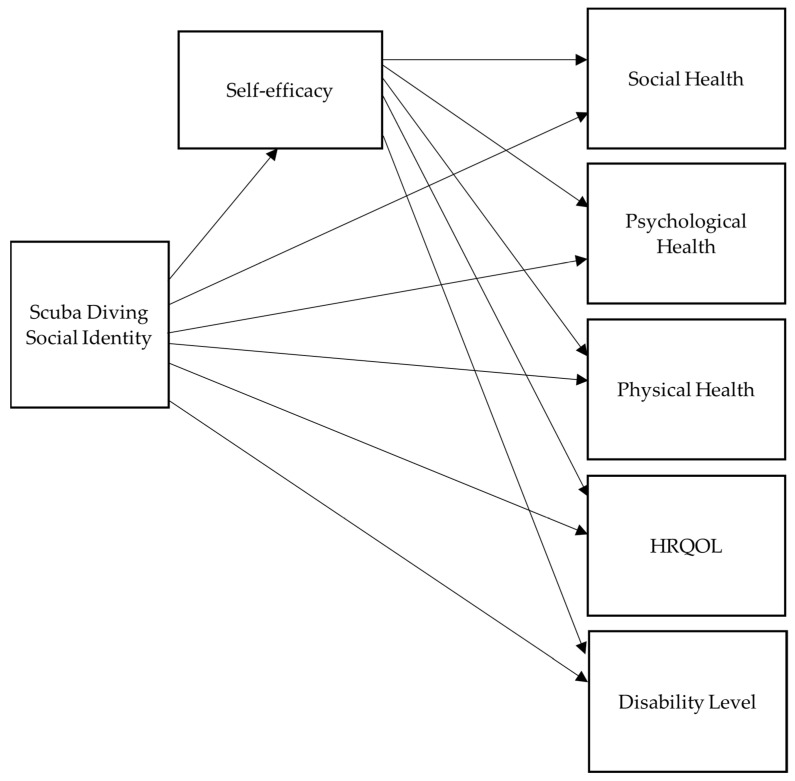
Hypothesized model or path diagram.

The statistical methods used for the analysis included a test of the model fit, summary of the descriptive statistics for each variable (mean, median, and standard deviation), correlation analysis via Pearson’s correlations, and path analysis with unstandardized and standardized results. The path analysis was used to test the directed dependencies across the variables. Figure 2 shows the model with the path diagram that was analyzed. Statistical analyses were conducted in *R* 4.1.2 [49] using the *lavaan* [50] package. 

### 2.4. Phase Two: Qualitative Phase

#### 2.4.1. Qualitative Data Collection

After the quantitative data analysis was completed, data collection for the qualitative phase followed. The quantitative results informed the sampling and data collection for the qualitative phase; survey participants who indicated willingness to participate in a follow-up interview were divided into 3 case study groups using principal component analysis (PCA) [41] scores and purposeful [38] sampling. The 3 case study groups consisted of high, low, and average scorers across the 5 dependent variables of social health, psychological health, physical health, HRQOL, and disability level. Thus, the 3 case study groups were comprised of participants who had the top 5 PCA scores, the lowest 5 PCA scores, and the 5 most average PCA scores. 

The qualitative data were collected using 1:1 semi-structured, audio- and video-recorded interviews. The Zoom [51] videoconference platform was used to conduct the interviews. The interview design process consisted of developing an interview protocol using an interview guide approach [52]. The interview questions were informed by the purpose of the explanatory mixed methods design and were formulated as a follow-up to the quantitative phase to learn more about the in-depth experiences of the participants. The interview questions were neutral and open-ended questions focused on participants’ descriptions of the relationships between 7 dimensions: (1) scuba diving social identity, (2) self-efficacy, (3) social health, (4) psychological health, (5) physical health, (6) HRQOL, and (7) disability level. The semi-structured nature of the interview allowed for probing questions to be asked, if necessary, to further explore the dimensions of interest. 

#### 2.4.2. Qualitative Data Analysis

Based on the social identity approach to health, 7 predetermined categories and 13 a priori codes guided the directed content analysis [53]. The 7 predetermined categories were: (1) scuba diving social identity, (2) self-efficacy, (3) social health, (4) psychological health, (5) physical health, (6) HRQOL, and (7) disability level. The 13 a priori codes under their respective predetermined categories are shown in Table 1. 

Following the directed content analysis [53], the researchers engaged in an inductive conventional content analysis [53] to develop new codes and categories. The final codes and categories were discerned using within-case [54] and cross-case [55] analyses with constant comparisons to determine similarities and differences across the 3 case study groups. The qualitative analysis helped develop themes to describe the relationships between scuba diving social identity and the levels of self-efficacy, social health, psychological health, physical health, HRQOL, and disability from the perspective of the participants. 

The trustworthiness strategies that were used include triangulation, member-checking, peer review, and reflexivity [56,57]. Two types of triangulation were used, participant and investigator. The participant triangulation consisted of dividing participants into 3 case study groups to ensure the representation of individuals with diverse quantitative results; the investigator triangulation consisted of having two researchers involved in the qualitative data analysis to avoid individual biases and ensure the accuracy of the data analysis and interpretation. The member checking consisted of returning to interview participants with a summary of the qualitative findings to check the accuracy of the results and for resonance with their experiences; here, 10 of the 15 interview participants took part in member checking, confirming the qualitative themes. To confirm the credibility and authenticity of the qualitative results, a second researcher peer reviewed 100% of the qualitative transcripts. Reflective journaling was used as a reflexivity strategy to acknowledge the researcher’s positionality throughout the qualitative phase of the study. 

### 2.5. Mixing of Quantitative and Qualitative Data

The study involved a final integration stage in which quantitative and qualitative results were mixed to achieve integrated conclusions. The results of the two phases were mixed to draw meta-inferences on how the qualitative results explained the quantitative results. A joint display was developed to present the mixed results, with a focus on how the qualitative results matched, elaborated, enhanced, or clarified the quantitative results [36]. 

## 3. Results

### 3.1. Quantitative Results

#### 3.1.1. Participants

The participants were recruited over a 3-month period, yielding a total of 103 surveys. For the path analysis, 78 observations were included in the model due to 21 data points being missing on the HRQOL variable, and 4 data points being missing on the disability level variable. Table 2 shows the demographic characteristics of the participants included in the path analysis. 

#### 3.1.2. Test of Model

**Figure 3:**
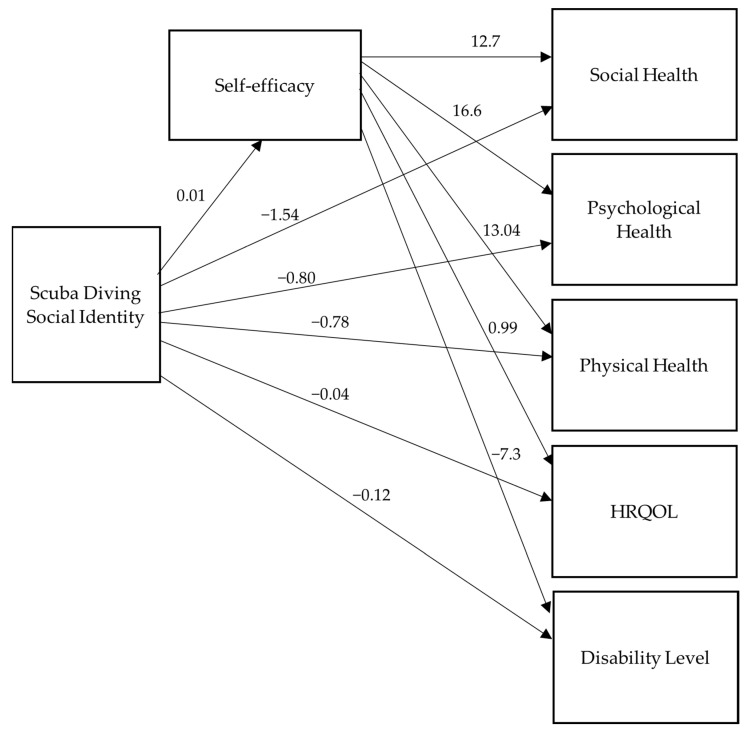
Unstandardized path model results.

**Figure 4:**
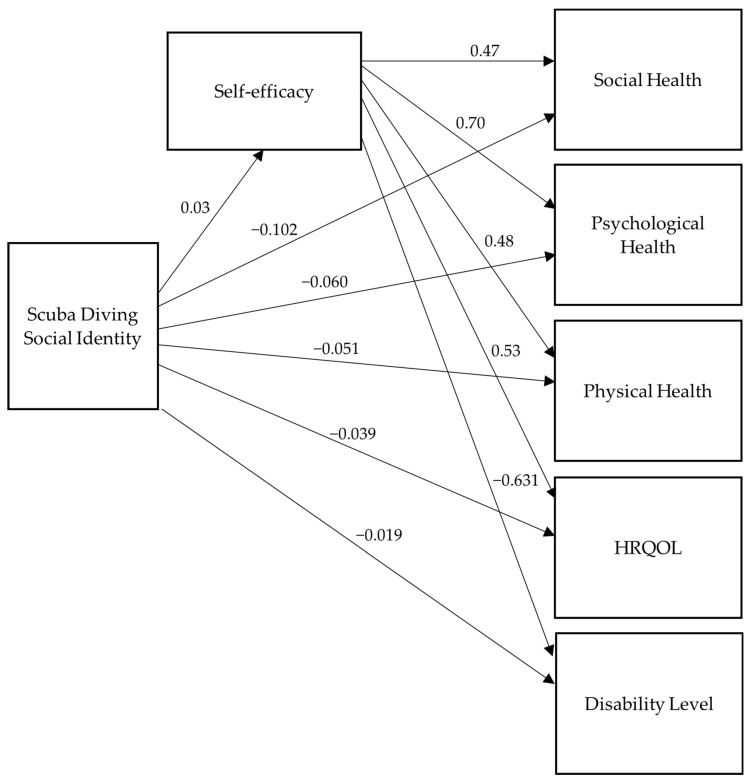
Standardized path model results.

The model had a model fit of CFI = TLI = 1.0 and RMSEA = SRMR = 0.0, with 0 *df*, indicating a just-identified model. Table 3 presents the mean, median, and standard deviation results for each variable in the model. See Figure 3 and Figure 4 for the results of the unstandardized and standardized path analyses. Table 4 shows the significance level of each path in the model. See Table 5 for the correlation coefficients between the variables, which were used to check for possible multicollinearity and correlations between the variables. Due to social identity not showing significance and its estimates not being in the predicted direction, the path model was re-analyzed using *z*-scores to standardize scaling differences across variables. The results of the path analysis with *z*-scores were still non-significant and in the opposite direction than predicted for scuba diving social identity. Due to scuba diving social identity having extreme negative skewness (−1.99) and leptokurtosis (6.96), range restriction was concluded. The social identity variable was log-transformed to minimize the influence of the negative skew; the path analysis with the log-transformed variable did not yield any differences in significance. However, the estimates were loading on the predicted direction. Thus, the range restriction on the scuba diving social identity variable yielded inconclusive statistical evidence for the absence or presence of the effects of this variable on other variables. 

### 3.2. Qualitative Results

#### 3.2.1. Participants

A total of 15 survey participants divided into 3 case study groups participated in the qualitative phase. Each case study group was composed of 5 participants representative of the (1) highest PCA, (2) lowest PCA, and (3) average PCA scorers across the 5 dependent variables of social health, psychological health, physical health, HRQOL, and disability level. Table 6 shows the demographic characteristics of the qualitative sample. The participant names were replaced with gender-neutral pseudonyms to maintain the confidentiality of the research participants. 

#### 3.2.2. Qualitative Themes

Six overarching themes were obtained from engaging in a directed content analysis followed by an inductive conventional content analysis. The within-case and across-case thematic analyses revealed that the 3 case study groups were homogenous in their answers, sharing the following 6 overarching themes: (1) being a scuba diver is a positive social identity that provides me with a sense of belonging, meaning, and purpose; (2) scuba diving has contributed positively to my social health through enhanced social relationships and a community that provides me with social support; (3) scuba diving has contributed positively to my psychological health by enhancing self-esteem, positive feelings, and relaxation and reducing symptoms of depression, anxiety, or post-traumatic stress disorder; (4) scuba diving has contributed positively to my physical health by offering physical activity or exercise, better mobility, relief from physical pain, and better sleep; (5) scuba diving has contributed positively to my self-efficacy by boosting my self-confidence; (6) scuba diving has contributed positively to the quality of my life. Table 7 presents the frequency results for the qualitative themes, categories, and codes. After the table, we provide a narrative description for each of the six themes.

**Theme** **1.** ***Being a scuba diver is a positive social identity that provides me with a sense of belonging, meaning, and purpose.***

The first theme that emerged related to participants’ perspectives of their scuba diving social identity. The participants reported that their scuba diving group membership provides them with a sense of belonging. For instance, Terry explained:

*Diving is a universal language, so even if you’re dealing with divers and there’s a language barrier, there’s still a common language that you share. And it ingratiates you with them, because you have shared experiences and it makes things easier, like we always identify as a group in some way.*

In addition to a sense of belonging, the participants narrated that their scuba diving identity provides them with meaning and purpose. Robin shared “*I think* (scuba) *it’s who I am, so it’s very extremely important…it’s given me a sense of purpose now*.” To illustrate how scuba gives them purpose, Nat explained *“It is an important part of my life…I’m advocating for other vets* (veterans) *to get out there…I want to not only be able to do more myself, but to bring up those others who can’t, and bring them along.*

**Theme** **2.** ***Scuba diving has contributed positively to my social health through enhanced social relationships and a community that provides me with social support.***

The participants reported perceiving that scuba diving has had an influence on improving their social skills and social relationships. Additionally, they explained that the scuba diving community provides them with social support. For example, when asked about the social influence of scuba diving, Arden articulated, *“Beforehand, I was so quiet and shy…I’m much more outgoing, much more willing to talk to people and go out of my way to talk to people...I became more extroverted…I’m more social now.*” Many participants described their developing friendships and experiencing camaraderie through scuba diving. For instance, Alex pointed out “*Just relationships that definitely have grown, especially with the new community I’m part of and that has definitely grown.* Uniformly, Eli said, *“You make new friends when you are going diving with them, or go to the diving clubs…the camaraderie, you know, of being with other divers…you make new friends, and they support you in what you’re doing*.”

**Theme** **3.** ***Scuba diving has contributed positively to my psychological health by enhancing self-esteem, positive feelings, and relaxation and reducing symptoms of depression, anxiety, or post-traumatic stress disorder (PTSD).***

The participants identified several mental health benefits from their participation in scuba diving. They predominantly mentioned that scuba diving has helped with boosting their self-esteem and promoting positive feelings and relaxation, as well as lessening depression, anxiety, and PTSD symptomatology. Regarding their self-esteem and experiencing positive emotions, Gael narrated the following:

*Self-esteem has improved because I’ve changed a lot from I can’t to I can. It actually made me love who I am as a person. For a long time I didn’t, because I can’t play basketball anymore, football out, frisbee, football is out, golf…I used to play softball…I was my kid’s soccer coach and trying to run after the ball and I’m eating it, so I mean, a lot of the world above the water is very negative for me. But then when I introduced the water, it made me very positive towards who I could be…because my activities in the water, now has made it to where I feel better about myself. It increased my self-esteem.*

Marion and Devin shared the following stories on how scuba diving has helped with relaxation, reducing their symptomatology of anxiety, depression, and PTSD:

*Honestly, I could say if I was not as active as a scuba diver, my depression and everything probably would have taken over, and just ruined my life….everybody’s always like…what does scuba diving do for you? and I’m like, well, it does a lot because a lot of people don’t understand when you’re underwater, the world just disappears, it’s a whole other world underwater, you don’t have all the busyness, you don’t have highway traffic, you don’t have the negative of the world. When you’re underwater, it’s freeing, it’s soothing…when I hear my bubbles while I’m diving and everything, it’s just calming.*

Devin

*I’m able to take the stresses of everyday life, the military life, the PTSD, and scuba diving is a way to let that out, to let that release, to let that become a different person. Mentally, it saved my life. Scuba diving has, it is kind of emotional to put it in words for me, but it has been able to…pull me off the edge. When I’m in the water, nobody’s threatening me, I don’t have to worry about killing-attack, or the PTSD, becoming overwhelmed with the noises…it has been kind of like a saving grace for me…it’s a sense of safety for me, so mentally it has greatly improved my ability to deal with my disabilities.*

Marion

**Theme** **4.** ***Scuba diving has contributed positively to my physical health by offering physical activity/exercise, better mobility, relief from physical pain, and better sleep.***

Overall, the participants reported that they considered scuba a great form of exercise or physical activity. Additionally, they discussed the physical health benefits derived from scuba diving, mainly in the areas of mobility, pain, and sleep. Addressing mobility, Win shared, “*It helps the mobility because under the water I get to experience a full range of motion that I wouldn’t be able to experience otherwise, and so it definitely helps with the mobility.”* Focusing on pain, Les pointed out that scuba, “*Has improved pain a lot…It’s a place where I can be pain free… There’s no pain, there’s freedom, I’m relaxed.*” Similarly, Eli expressed:

*Muscle spasms can be painful, and diving helps to release, relax the muscles and loosen the legs, so there’s not as many spasms and it’s not as painful. The pain and the spasticity in my legs and arms almost goes to zero under the water…And after I dive, I’m, you know, several hours of relief.*

Addressing sleep, Robin shared “*I have an extreme hard time sleeping, I’ve got to take medicine to try to sleep at night. And noticing after diving, I’ll feel a whole lot better, I’ll be able to sleep, I don’t have to take medicine.”*

**Theme** **5.** ***Scuba diving has contributed positively to my self-efficacy by boosting my self-confidence.***

The participants reported an influence of scuba diving on their self-confidence and on their beliefs in their ability to complete new tasks and challenges. For example, Briar expressed:

*(Scuba diving) It’s a challenge, and each time I can get in and do it, it helps me realize that you know, even though I’m injured, I can still get out and do these things, so it helps me say, you know, -hey, I’m doing good, I’m doing better; I can do this-. I may need a little help, but I can still do it…it probably has given me more confidence because, -hey I can do this-, and so I see myself as able to do more. And so, I can be more of an overcomer.*

Alex shared how they felt more confident about earning more scuba certifications. Similarly, Terry described, *“You build a lot of confidence in learning the skill and then executing the skill in a high stakes environment…I see myself as more confident individual because I’m able to do new things and take on new challenges.”*

**Theme** **6.** ***Scuba diving has contributed positively to the quality of my life.***

The last theme that emerged from the qualitative data analysis was the positive influence of scuba diving on life quality among the interviewed scuba divers with physical impairments. Sal shared, *“It has improved the quality of my life because it has given me new experiences, you know, and new ways of seeing the world differently.”* Morgan described better quality of life by means of discovering a new favorite activity. Morgan said, *“Imagine discovering your most favorite activity, after years of not having a favorite activity.”* Kim provided an overall description of the different areas of their life that diving has contributed to:

*For me the quality life is what I’m looking for and scuba diving helps my quality of life, and like what we talked about, emotionally, physically, and then like I said, you have other aspects, like social jumps into it or recreational…it’s really neat that you can find something that has all of those. I used to be a wrestler and a boxer and it was all physical, I can’t do those as much anymore, but now, I can do something that helps my every day to day life trying to, and it may not help me all the time…when I’m not diving, but it will help me when I’m diving, and it may not help me physically when I’m not diving but it helps me emotionally or socially.*

### 3.3. Mixed Results

Table 8 compares the quantitative and qualitative results. Table 9 shows a joint display summarizing the two datasets and the meta-inferences from the data integration process. The two tables address the relationships between scuba diving social identity, self-efficacy, social health, psychological health, physical health, HRQOL, and disability level among scuba divers with physical impairments. 

## 4. Discussion

The explanatory sequential mixed methods research presented here took a social identity approach to health to investigate the relationships between scuba diving social identity, self-efficacy, social health, psychological health, physical health, HRQOL, and disability level among people with physical impairments. At first, the quantitative and qualitative results appeared to contradict each other on the influence of scuba diving social identity on self-efficacy, social health, psychological health, physical health, HRQOL, and disability level. However, a further analysis of the quantitative data led us to determine that the survey data results on scuba diving social identity are inconclusive due to the range restriction on the scuba diving social identity variable. The survey data in the sample were not sufficiently strong for the social identity variable, which had extreme negative skewness and leptokurtosis, so we could not conclude that the path analysis and correlation results indicated evidence of the absence of correlations or effects. The qualitative data provided insights about the type of social identity scuba divers with physical impairments considered scuba diving to be, and its influence on their self-efficacy, social health, psychological health, physical health, HRQOL, and disability level. Notwithstanding the participants being divided into 3 case study groups based on their survey results (i.e., low, average, and high scorers), the within-case and across-case analyses of the interviews revealed that the qualitative data from the 3 case study groups were homogenous. The participants of the 3 case study groups shared very similar answers and experiences, suggesting that the participants had strong perceptions of the positive influence that scuba diving has had on their self-efficacy, social health, psychological health, physical health, HRQOL, and disability level. 

The participants of the quantitative sample had extremely high scuba diving social identification, resulting in a lack of variability to establish relationships for the social identity variable. More data would be required to yield more conclusive results on the effects of scuba diving social identity on social health, psychological health, physical health, HRQOL, and disability level among people with physical impairments. We recommend follow-up analyses with larger sample sizes and more variability in the samples; the samples should include more participants who possess low and medium scuba diving social identification, in addition to those who possess high scuba diving social identification. We also recommend that future studies compare people who are scuba divers and people who are not scuba divers. To further understand the mechanisms linking social identity to health, Hausser and colleagues [58] recommended using a group-level operationalization process of shared identity as a predictor of health in addition to the individual’s identification; these authors [58] noted that testing group-level mechanisms while using individual identification as the predictor could potentially underestimate the strength of the proposed relationships. They recommended that future studies combine three different aspects of social identity, including individual identification, group identification, and perceived group identification. Additionally, they proposed a social identity model with a two-step serial mediation of the effect of group identification on health via mutual social support and collective self-efficacy. Lastly, they recommended that future research look at cross-level interactions between group-level mechanisms (i.e., mutual support and collective self-efficacy) and individual-level mechanisms (i.e., attribution and appraisal processes) [58]. Therefore, more possibilities remain for the exploration of the influence of social identity on the health of scuba divers with physical impairments.

The first theme of the qualitative strand proposes that scuba diving social identity is a positive social identity that provides a sense of belonging, meaning, and purpose to scuba divers with physical impairments; this result aligns scuba diving social identity to the social cure identities described in the existing literature on the social identity approach to health [14,15,59,60]. Additionally, the rest of the qualitative themes suggest that scuba diving has a positive influence on the self-efficacy, social health, psychological health, physical health, and quality of life among people with physical impairments. The influence of scuba diving on self-efficacy was described by participants as scuba boosting their self-confidence and aiding in their development of a can-do attitude. The previous available evidence discussed scuba diving as a means to building confidence and independence [21,61]. The influence of scuba on social health was described by participants as promoting improved social relationships and having a community that provides them with social support; these results concurred with previous reports on scuba divers with physical impairments reporting an improved scope and quality of social interactions [22], enhanced opportunities for socialization and connection [32], and more social support [23,32]. The influence of scuba diving on psychological health involved participants reporting improved self-esteem; positive feelings; relaxation; and reduced symptomatology of anxiety, depression, and PTSD. Previous research has documented similar results, including improved self-esteem [22], self-concept [21,23,24,32], and alleviation in the symptomatology of anxiety [28], depression [28], and PTSD [62]. The influence of scuba diving on physical health was reported by participants mostly in terms of more physical activity, better mobility, relief from physical pain, and better sleep. The physical health outcomes previously reported from scuba diving among people with physical impairments included chronic pain relief [22,23,24,25,26,27,28], reduced spasticity and frequency of muscle spasms [25,26], increased pulmonary vital capacity and efficiency of the respiratory system [22,26], improved circulation and body strength [34], increased motor skills [22], and increased general physical ability [31].

The participants in our study reported that scuba contributed positively to the quality of their lives. Our results match the previous available evidence that suggested that scuba diving can contribute positively to the quality of life of scuba divers with physical impairments [23,28,32,63]. The study with the largest sample size (*n* = 64) prior to ours found that the quality of life of scuba divers with physical impairments was higher in the areas of mental health and social performance compared to non-scuba divers, independently of the degree of their disability [63]. We recommend that future studies explore the specific mechanisms of scuba diving that may contribute to these health and quality of life outcomes. The qualitative results of our study support previous evidence on the positive effects of a shared social identity on health and quality of life [59,64]. In their systematic review and meta-analysis of 27 social identification-building interventions (*n* = 2230), Steffens and colleagues [59] found that group-relevant decision-making and therapy interventions had relatively large effects, while those involving shared activities had relatively small effects. Our study focused on scuba diving as a shared activity rather than as a therapy intervention. We recommend rehabilitation professionals consider intentionally adding group identification interventions, including scuba diving, to their therapy programs and study the results of their interventions. We also recommend the future exploration of scuba diving as a biopsychosocial and sociopsychobio therapy intervention. 

The results of the cross-sectional survey indicated that self-efficacy was a strong predictor of social health, psychological health, physical health, HRQOL, and disability level among scuba divers with physical impairments. Similarly, the qualitative data showed that participants considered that scuba diving positively influenced their self-efficacy; most participants’ descriptions consisted of them explaining how scuba diving has boosted their self-confidence, both within scuba diving and across other life activities. The meta-inferences derived from data integration of quantitative and qualitative data strands contributed valuable insights into the role of scuba diving on self-efficacy and the role of self-efficacy on health, HRQOL, and disability level. The previous evidence has shown that high self-efficacy is related with higher QOL, improved health outcomes, less secondary health conditions (e.g., anxiety and depression), reduced disability, and increased disability self-management among people with physical impairments [64,65,66,67,68,69,70]. We recommend that rehabilitation professionals target self-efficacy during their interventions. We also recommend that future studies explore activity-specific self-efficacy and collective self-efficacy in addition to general self-efficacy. Additionally, we recommend the exploration of the specific mechanisms of scuba diving that may lead to improved self-efficacy, physical health, psychological health, social health, and HRQOL among people with physical impairments. 

This study supports self-efficacy as a primary contributory factor to the health and quality of life of people with physical impairments. The implications for practice include healthcare and rehabilitation professionals focusing on interventions and strategies that provide opportunities to develop, maintain, or improve their self-efficacy. A focus on self-efficacy may improve the clinical outcomes and long-term management of disability among people living with physical impairments. Through self-efficacy, people with physical impairments may gain additional skills and the ability to control and overcome circumstances or challenges that they may face in their lives. 

This study creates awareness regarding scuba diving as a potential therapeutic and rehabilitation modality. Our project constitutes exploratory work on the influence of scuba diving on health domains and quality of life for people with physical impairments. People with physical impairments, healthcare providers, and researchers who may be interested are encouraged to further explore the therapeutic benefits of scuba diving. We also recommend the exploration of diagnostic-specific barriers, risk factors, and safety considerations. The researchers noticed that having a military background was a common and unanticipated characteristic of the qualitative sample; 11 of the 15 interviewed participants self-reported being veterans. We recommend further studies exclusively explore the benefits of scuba diving for veterans, a population with complex medical needs. Previous papers have focused on the benefits of scuba diving for this group, with promising findings on both their medical and psychological needs [28,62]. 

## 5. Conclusions

The present research intended to advance the social identity approach to health by testing the relationships between scuba diving social identity and self-efficacy, social health, psychological health, physical health, HRQOL, and disability level among people with physical impairments. The quantitative strand yielded inconclusive evidence on the influence of scuba diving social identity due to range restrictions on the scuba diving social identity variable. More research is needed to reconcile the gaps in the quantitative data strand and to shed more light on the topic. The results of the cross-sectional survey indicated that self-efficacy was a strong predictor of social health, psychological health, physical health, HRQOL, and disability level among scuba divers with physical impairments. Our results contribute to the available evidence on the influence of self-efficacy on health, HRQOL, and disability management. We recommend healthcare and rehabilitation professionals target self-efficacy as a means to improve the physical health, psychological health, social health, and HRQOL of their clients. The qualitative results of our study support the previous evidence on the positive effects of a shared social identity on health and quality of life; the interview participants reported that scuba diving contributed positively to their self-efficacy, social health, psychological health, physical health, and HRQOL. This study may serve as motivation for healthcare professionals and researchers to consider the exploration of scuba diving as both a health promotion recreational activity and rehabilitation modality. 

## Figures and Tables

**Table 1:** The qualitative analysis of a priori codes.

Predetermined Category	A Priori Codes
Scuba diving social identity	Sense of belonging
	Positive distinctiveness
	Emotional significance
Social health	Personal relationships
	Social support
	Sexual activity
Psychological health	Bodily image and appearance
	Positive vs. negative feelings
	Self-esteem
	Thinking, learning, memory, and concentration
Physical health	Energy and fatigue
	Pain and discomfort
	Sleep and rest

**Table 2:** Demographic characteristics of quantitative samples (*n* = 78).

Characteristic	*N*	(%)
Impairment Category		
Spinal Cord Injury	20	25.6%
Acquired/Traumatic Brain Injury	12	15.4%
Amputation or Limb Loss	6	7.7%
Degenerative Disc Disease	5	6.4%
Arthritis	4	5.1%
Chronic Back Pain	4	5.1%
Multiple Sclerosis	4	5.1%
Fibromyalgia	3	3.8%
Joint Reconstruction or Replacement	3	3.8%
Sensory Impairment	3	3.8%
Muscular Dystrophy	2	2.6%
Spinal Stenosis	2	2.6%
Amyotrophic Lateral Sclerosis	1	1.28%
Cerebral Palsy	1	1.28%
Clonus	1	1.28%
De Quervain’s Syndrome	1	1.28%
Lupus	1	1.28%
Neuro-Sweet Disease	1	1.28%
Poliomyelitis	1	1.28%
Polymyositis	1	1.28%
Scoliosis	1	1.28%
Transverse Myelitis	1	1.28%
Impairment Onset		
As an adult (19+)	63	80.8%
Before age 18	11	5.1%
Congenital	4	14.1%
Gender		
Male	54	69.2%
Female	24	30.8%
Non-binary/Third Gender	0	0.0%
Race/Ethnicity		
White	63	80.8%
Hispanic or Latinx	6	7.7%
Multiracial	4	5.1%
Black or African American	4	5.1%
American Indian or Alaskan Native	1	1.3%
Age		
20–29	1	1.3%
30–39	14	17.9%
40–49	25	32.0%
50–59	26	33.3%
60–69	11	14.1%
70–79	1	1.3%
Length of Scuba Diving Group Membership		
<1 year	4	5.1%
1–3 years	17	21.8%
4–6 years	19	24.3%
7–9 years	10	12.9%
10+ years	28	35.9%
Diving Level		
Beginner	13	16.7%
Intermediate	24	30.7%
Advanced	24	30.7%
Expert	17	21.8%
Logged Dives per Year		
1–5	19	24.3%
6–10	9	11.5%
11–15	10	12.8%
16–20	11	14.1%
21–25	2	2.5%
26–30	6	7.7%
>30	20	25.6%
NA	1	1.3%

**Table 3:** Mean, median, and standard deviation values of the model variables.

Variable	Mean	Median	SD
Scuba diving social identity	6.07	7.00	1.41
Self-efficacy	3.98	4.00	0.72
Social health	59.74	58.33	19.98
Psychological health	64.51	63.75	18.46
Physical health	53.90	52.08	21.41
HRQOL	2.81	4.00	1.45
Disability level	13.84	12.00	8.85

**Table 4:** Significance level of each path in the model.

Path	*z*-Value	*p*
Scuba social identity to self-efficacy	0.303	0.762
Scuba social identity to social health	−1.032	0.302
Scuba social identity to psychological health	−0.738	0.461
Scuba social identity to physical health	−0.514	0.607
Scuba social identity to HRQOL	−0.406	0.685
Scuba social identity to disability level	−0.214	0.830
Self-efficacy to social health	4.790	<0.001
Self-efficacy to psychological health	8.648	<0.001
Self-efficacy to physical health	4.853	<0.001
Self-efficacy to HRQOL	5.586	<0.001
Self-efficacy to disability level	−7.186	<0.001

Note: Range restriction impaired conclusive evidence for scuba social identity variable.

**Table 5:** Pearson’s correlation coefficients.

	Self-Efficacy		Scuba Diving Social Identity	
	*r*	*p*	*r*	*p*
Social Health	0.50	<0.001	−0.12	0.2171
Psychological health	0.70	<0.001	−0.07	0.5089
Physical health	0.45	<0.001	−0.04	0.7055
HRQOL	0.54	<0.001	−0.03	0.8132
Disability level	−0.60	<0.001	−0.09	0.3739
Self-efficacy			−0.01	0.8939

**Table 6:** Demographic characteristics of the qualitative sample (*n* = 15).

Characteristic	*n*	(%)
Impairment Category		
Spinal Cord Injury	5	33.3%
Acquired/Traumatic Brain Injury	3	20.0%
Chronic Back Pain	2	13.3%
Arthritis	1	6.7%
Fibromyalgia	1	6.7%
Joint Reconstruction or Replacement	1	6.7%
Muscular Dystrophy	1	6.7%
Polymyositis	1	6.7%
Impairment Onset		
As an adult (age 19+)	13	86.6%
Before age 18	1	6.7%
Congenital	1	6.7%
Gender		
Male	12	80%
Female	3	20%
Non-binary/Third Gender	0	0.0%
Race/Ethnicity		
White	10	66.6%
Hispanic or Latinx	2	13.3%
Multiracial	2	13.3%
Black or African American	1	6.7%
Age		
20–29	1	6.7%
30–39	2	13.3%
40–49	6	40.0%
50–59	5	33.3%
60–69	1	6.7%
Length of Scuba Diving Group Membership		
<1 year	1	6.7%
1–3 years	2	13.3%
4–6 years	3	20.0%
7–9 years	4	26.7%
10+ years	5	33.3%
Diving Level		
Beginner	1	6.7%
Intermediate	7	46.6%
Advanced	3	20.0%
Expert	4	26.7%
Logged Dives per Year		
1–5	4	26.7%
6–10	4	26.7%
11–15	0	0.0%
16–20	1	6.7%
21–25	1	6.7%
26–30	2	13.3%
>30	3	20.0%

**Table 7:** Frequency results for qualitative themes, categories, and codes (*n* = 15).

Themes	*Freq.	Categories	*Freq.	Codes	*Freq.
Theme 1: Being a scuba diver is a positive social identity that provides me with a sense of belonging, meaning, and purpose.	15	Scuba diving social identity	15	*Deductive*	
				Sense of belonging	14
				Positive distinctiveness	11
				Emotional significance	14
				*Inductive*	
				Meaningful identity	14
				Purposeful identity	14
				Positive identity	15
Theme 2: Scuba diving has contributed positively to my social health through enhanced social relationships and a community that provides me with social support.	15	Social health	15	*Deductive*	
				Personal relationships	14
				Social support	13
				Sexual activity	0
				*Inductive*	
				Community	14
Theme 3: Scuba diving has contributed positively to my psychological health by enhancing self-esteem, positive feelings, and relaxation; and reducing symptoms of depression, anxiety, and/or post-traumatic stress disorder (PTSD).	15	Psychological health	15	*Deductive*	
				Bodily image & appearance	6
				Positive feelings	15
				Self-esteem	13
				Thinking, learning, memory, and concentration	7
				*Inductive*	
				Relaxation	14
				Anxiety and depression	11
				PTSD	8
Theme 4: Scuba diving has contributed positively to my physical health by offering physical activity/exercise, better mobility, relief from physical pain, and better sleep	15	Physical health	15	*Deductive*	
				Energy and fatigue	14
				Pain and discomfort	12
				Sleep and rest	11
				*Inductive*	
				Physical activity/exercise	14
				Improved mobility	11
Theme 5: Scuba diving has contributed positively to my self-efficacy by boosting my self-confidence.	14	Self-efficacy	14	*Inductive*	
				Self-confidence	13
				Accomplishments & goals	8
Theme 6: Scuba diving has contributed positively to the quality of my life.	14	Health-related quality of life	12	*Inductive*	
				Positive influence on different life domains.	14

* Frequency.

**Table 8:** Comparison of quantitative and qualitative results.

Dimension	QUAN Results	QUAL Results	Mixed-Methods Comparison
Influence of scuba diving social identity on self-efficacy	Scuba diving social identity does not influence self-efficacy (r = −0.01, *p* = 0.8939, β = 0.01, *p* = 0.762)	Themes 1 & 5 Scuba diving social identity influences self-efficacy	Discrepancy between quantitative and qualitative results
Influence of scuba diving social identity on social health	Scuba diving social identity does not influence social health (r = −0.12, *p* = 0.2171, β = −1.54, *p* = 0.302)	Themes 1 & 2 Scuba diving social identity influences social health	Discrepancy between quantitative and qualitative results
Influence of scuba diving social identity on psychological health	Scuba diving social identity does not influence psychological health (r = −0.07, *p* = 0.5089, β = −0.80, *p* = 0.461)	Themes 1 & 3 Scuba diving social identity influences psychological health	Discrepancy between quantitative and qualitative results
Influence of scuba diving social identity on physical health	Scuba diving social identity does not influence physical health (r = −0.04, *p* = 0.7055, β = −0.78, *p* = 0.607)	Themes 1 & 4 Scuba diving social identity influences physical health	Discrepancy between quantitative and qualitative results
Influence of scuba diving social identity on HRQOL	Scuba diving social identity does not influence HRQOL (r = −0.03, *p* = 0.8132, β = −0.04, *p* = 0.685)	Themes 1 & 6 Scuba diving social identity influences HRQOL	Discrepancy between quantitative and qualitative results
Influence of scuba diving social identity on disability level	Scuba diving social identity does not influence disability level (r = −0.09, *p* = 0.3729, β = −0.12, *p* = 0.830)	Themes 1, 2, 3, 4 Scuba diving social identity influences disability level	Discrepancy between quantitative and qualitative results

**Table 9:** Joint display with mixed methods results on the relationships between scuba diving social identity, self-efficacy, social health, psychological health, physical health, HRQOL, and disability level among people with physical impairments.

Quantitative Findings	Qualitative Findings	Meta-Inferences
Pearson’s correlation coefficients from the sample did not show any correlations between scuba diving social identity and social health (r = −0.12, *p* = 0.2171), psychological health (−0.07, *p* = 0.5089), physical health (r = −0.04, *p* = 0.7055), HRQOL (r = −0.03, *p =* −0.8132), disability level (r = −0.09, *p* = 0.3729), or self-efficacy (−0.01, *p* = 0.8939) The path analysis from the sample did not show scuba diving social identity as a predictor of social health (β= −1.54, *p* = 0.302), psychological health (β= −0.80, *p* = 0.461), physical health (β = −0.78, *p* = 0.607), HRQOL (β = −0.04, *p* = 0.685), or disability level (β = −0.12, *p* = 0.830) Pearson’s correlation coefficients from the sample showed strong positive correlations between self-efficacy and social health (r = 0.50, *p* = 0.000), psychological health (r = 0.70, *p* = 0.000), and HRQOL (r = 0.54, *p* = 0.000). Moderate positive correlations were found between self-efficacy and physical health (r = 0.45, *p* = 0.000). Additionally, there was a strong negative correlation between self-efficacy and disability level (r = −0.60, *p* = 0.000). The path analysis from the sample showed that self-efficacy significantly predicted social health (β = 12.71, *p* = 0.000), psychological health (β = 16.60, *p* = 0.000), physical health (β = 13.04, *p* = 0.000), HRQOL (β = 0.99, *p* = 0.000), and disability level (β = −7.37, *p* = 0.000)	Theme 1: Being a scuba diver is a positive social identity that provides me with a sense of belonging, meaning, and purpose. *“I have a connection with other scuba divers, and with sea-related material like everyone in this community.”* Alex *“Scuba has had a huge impact on my life. I started scuba diving when I was 14. I got certified with my dad. And then, last year I got certified up to dive master and now, that’s actually what I do for a living…I’m a dive guide, like it’s really helped me.”* Arden *“One of my goals as part of my mission statement is to help other combat veterans reconnect to life through the outdoors. And one of my goals is to be able to pour into other combat veterans’ lives. I have been able to do that through scuba.”* Briar Theme 2: Scuba diving has contributed positively to my social health through enhanced social relationships and a community that provides me with social support. *“It helps you gain friendships, having camaraderie, the ability to meet new people, and when you find a group of people that have the same interest, it’s usually easier to become, have a friendship started. When I dive, I’m going to be in a pretty darn good mood, I’m pretty happy, so that helps you socially with your spouse, that helps you socially with your kids, or even friends.” Kim* *“Within the scuba diving community, everybody’s super supportive, it’s across the board, everybody respects you and you respect everybody, and it’s just a good community.”* Devin *“When I’m diving, I’m more, I guess you could say more friendlier to the others around me so, I’m more receptive to listening to people and I would open up some more.”* Robin Theme 3: Scuba diving has contributed positively to my psychological health by enhancing self-esteem, positive feelings, and relaxation; and reducing symptoms of depression, anxiety, or post-traumatic stress disorder (PTSD). “*Every time I get a new certification, it gives me more self-esteem, it gives me more pride*.” Nat “*You’re like there in the moment and enjoying it. For me, it becomes almost euphoric and there’s a high that exists for like a week after diving, where the decompression of doing the activity bleeds over into every aspect of my life in a positive way. More so, than other things that I do.”* Terry *“When I come out the water after diving, I’m not depressed, I’m not unhappy, I’m thrilled that I’m doing it, I know I’m in the place where I should be at. For us, for the PTSD and everything like that I think it’s one of the best therapies out there, the best medicine for this kind of a disease.”* Robin Theme 4: Scuba diving has contributed positively to my physical health by offering physical activity or exercise, better mobility, relief from physical pain, and better sleep. *“It helps me stay active…I wanted to lose weight when I started, when I first started receiving the training for the certification, and I was able to do it because it got me moving.”* Sal *“Scuba has helped decrease my pain…it physically makes me more tired, so I can sleep better and gets rid of my pain, so I can sleep better…it helps me stay more mobile. If I hurt, I don’t want to move. I get in the water, I don’t hurt, I move. Movement helps keep me active, scuba diving helps keep me active. Being active helps out my heart, my muscles, my joints*.” Briar “*I can feel like just the ability to move more fluidly in the water. Moving in those ways doesn’t hurt, so if it doesn’t hurt to move, mobility is improved, just hands down.”* Terry Theme 5: Scuba diving has contributed positively to my self-efficacy by boosting my self-confidence. *“Scuba has really deeply ignited confidence that was lost, it absolutely has influenced my self-confidence. Because of scuba diving, I did apply for a retreat and that’s not something I ever would have done, but scuba diving opened up that door for me to wanna go do that. As far as other things, I bike now, diving has helped me improve that to where now I can go bike riding.”* Marion *“It has influenced my confidence in that it goes back to that aspect of you know, if you can learn to do this, you can learn to do whatever else you come across in life.”* Eli *“It kind of goes back to you know, not knowing it’s possible, and now we know we can do this, let’s do more. After we dove a few times, my goal was to get 100 dives. When I hit 100 dives, I wanted to hit 200 dives. Now, I’m at 217; my next goal is 300.”* Morgan Theme 6: Scuba diving has contributed positively to the quality of my life. *“It’s expanded my life experiences, it’s expanded my social circle, and it’s given me an outdoor recreation activity that is new, and it’s something that I enjoy. It’s very much improved the quality of my life, very much.”* Win *“It influences me on my quality of life, my quality of life has improved because I’ve improved through my self-esteem because of being able to do different things with diving and learning.”* Gael *“Scuba is part of my life; it is a lifeline. It has influenced the quality of my life because it has left a positive influence, a positive striving influence, exciting influence, and lastly, it is something that I can do and enjoy.”* Les	Data on social identity were not converged due to range restrictions on the quantitative strand on the scuba social identity variable. Range restrictions affect correlations and predictions, meaning the scuba diving social identity results for the correlation and path analyses may have underestimated the relationships between scuba social identity and the rest of the variables. The social identity approach to health, previous evidence on this theory, and the qualitative results support social identity as a predictor of health and HRQOL. Thus, we recommend future studies with individuals who have greater variability in their degree of scuba diving social identification. The quantitative results found self-efficacy as a strong predictor of social health, psychological health, physical health, HRQOL, and disability level among scuba divers with physical disabilities. The qualitative results indicated that participants perceived scuba diving to be a strong contributor to their self-efficacy; they attributed part of their enhanced self-efficacy to scuba diving. The qualitative results added meaningful insight and depth to the understanding of the dependent variables. The participants described scuba diving as a positive contributor to their social health, psychological health, physical health, and HRQOL. Further research on the mechanisms of scuba diving that may contribute to these outcomes would be worthwhile.

## Data Availability

The data are unavailable due to privacy or ethical restrictions.

## References

[1] Jesus T.S., Landry M.D., Hoenig H. (2019). Global Need for Physical Rehabilitation: Systematic Analysis from the Global Burden of Disease Study 2017. Int. J. Environ. Res. Public Health.

[2] Reichard A., Stolzle H., Fox M.H. (2011). Health disparities among adults with physical disabilities or cognitive limitations compared to individuals with no disabilities in the United States. Disabil. Health J..

[3] Krahn G.L., Walker D.K., Correa-De-Araujo R. (2015). Persons with Disabilities as an Unrecognized Health Disparity Population. Am. J. Public Health.

[4] Zack M. (2013). Health-Related Quality of Life-United States, 2006 and 2010. MMWR Surveill. Summ..

[5] Keramat S.A., Ahammed B., Mohammed A., Seidu A.-A., Farjana F., Hashmi R., Ahmad K., Haque R., Ahmed S., Ali M.A. (2022). Disability, physical activity, and health-related quality of life in Australian adults: An investigation using 19 waves of a longitudinal cohort. PLoS ONE.

[6] Krops L.A., Jaarsma E.A., Dijkstra P.U., Geertzen J.H.B., Dekker R. (2017). Health Related Quality of Life in a Dutch Rehabilitation Population: Reference Values and the Effect of Physical Activity. PLoS ONE.

[7] Lima-Castro S., Blanco V., Otero P., López L., Vázquez F.L. (2020). Health-related quality of life among persons with physical disabilities: A systematic review and meta-analysis. Rev. Iberoam. Psicol. Salud.

[8] Jetten J., Haslam S.A., Cruwys T., Greenaway K.H., Haslam C., Steffens N.K. (2017). Advancing the social identity approach to health and well-being: Progressing the social cure research agenda. Eur. J. Soc. Psychol..

[9] Haslam C., Jetten J., Cruwys T., Dingle G.A., Haslam S.A. (2018). The New Psychology of Health: Unlocking the Social Cure.

[10] Haslam S.A., Jetten J., Postmes T., Haslam C. (2009). Social Identity, Health and Well-Being: An Emerging Agenda for Applied Psychology. Appl. Psychol..

[11] Engell G.L. (1977). The need for a new medical model: A challenge for biomedicine. Sci. New Ser..

[12] Haslam S.A., Haslam C., Jetten J., Cruwys T., Bentley S. (2019). Group life shapes the psychology and biology of health: The case for a sociopsychobio model. Soc. Pers. Psychol. Compass.

[13] Muldoon O.T., Walsh R.S., Curtain M., Crawley L., Kinsella E.L. (2019). Social cure and social curse: Social identity resources and adjustment to acquired brain injury. Eur. J. Soc. Psychol..

[14] Mertens N., Boen F., Steffens N.K., Haslam S.A., Bruner M., Barker J.B., Slater M.J., Fransen K. (2021). Harnessing the power of ‘us’: A randomized wait-list controlled trial of the 5R shared leadership development program (5RS) in basketball teams. Psychol. Sport Exerc..

[15] Kinsella E.L., Muldoon O.T., Fortune D.G., Haslam C. (2018). Collective influences on individual functioning: Multiple group memberships, self-regulation, and depression after acquired brain injury. Neuropsychol. Rehabil..

[16] Tajfel H., Turner J.C., Austin W.G., Worchel S. (1979). An integrative theory of inergroup conflict. The Social Psychology of Intergroup Relations.

[17] Willer D., Turner J.C., Hogg M.A., Oakes P.J., Reicher S.D., Wetherell M.S. (1987). Rediscovering the Social Group: A Self-Categorization Theory.

[18] Walker G.J., Kleiber D.A., Mannell R.C. (2019). A Social Psychology of Leisure.

[19] Kleiber D. (1999). Leisure Experience and Human Development: A Dialectical Interpretation (Lives in Context).

[20] Kelly J.R. (1983). Leisure Identities and Interactions.

[21] Naumann K., Kernot J., Parfitt G., Gower B., Winsor A., Davison K. (2021). What are the effects of scuba diving-based interventions for clients with neurological disability, autism or intellectual disability? A systematic review. Diving Hyperb. Med. J..

[22] Henrykowska G., Soin J., Siermontowski P. (2021). Scuba Diving as a Form of Rehabilitation for People with Physical Disabilities. Int. J. Environ. Res. Public Health.

[23] Aganovic Z. (2019). Effects of scuba diving programmed classes on Bosnian war veterans with amputations. Int. J. Sport Exerc. Train. Sci..

[24] Williamson J.A., McDonald F.W., Galligan E.A., Baker P.G. (1984). Selection and training of disabled persons for scuba-diving: Medical and psychological aspects. Med. J. Aust..

[25] Haydn T., Brenneis C., Schmutzhard J., Gerstenbrand F., Saltuan L., Schmutzhard E. (2007). Scuba diving—A therapeutic option for patients with paraplegia. Neuropsychiatrie.

[26] Novak H.F., Ladurner G. (1999). Scuba diving as a rehabilitation approach in paraplegia. Rehabilitation.

[27] Blumhorst E., Kono S., Cave J. (2020). An Exploratory Study of Adaptive Scuba Diving’s Effects on Psychological Well-Being among Military Veterans. Ther. Recreat. J..

[28] Morgan A., Sinclair H., Tan A., Thomas E., Castle R. (2019). Can scuba diving offer therapeutic benefit to military veterans experiencing physical and psychological injuries as a result of combat? A service evaluation of Deptherapy UK. Disabil. Rehabil..

[29] Beneton F., Michoud G., Coulange M., Laine N., Ramdani C., Borgnetta M., Breton P., Guieu R., Rostain J.C., Trousselard M. (2017). Recreational Diving Practice for Stress Management: An Exploratory Trial. Front. Psychol..

[30] Cracknell D., White M.P., Pahl S., Nichols W.J., Depledge M.H. (2016). Marine Biota and Psychological Well-Being: A preliminary examination of dose–response effects in an aquarium setting. Environ. Behav..

[31] Graczyk D. (2010). Diving and autonomic cardiovascular system regulation in persons with paraplegia. Med. Rehabil..

[32] Carin-Levy G., Jones D. (2007). Psychosocial aspects of scuba diving for people with physical disabilities: An occupational science perspective. Can. J. Occup. Ther..

[33] Madorsky J.G., Madorsky A.G. (1988). Scuba diving: Taking the wheelchair out of wheelchair sports. Arch. Phys. Med. Rehabil..

[34] Boyd L.A. (1972). Scuba provides expression. J. Rehabil..

[35] Dimmock K. (2009). Finding comfort in adventure: Experiences of recreational SCUBA divers. Leis. Stud..

[36] Creswell J.W., Plano Clark V.L. (2018). Designing and Conducting Mixed Methods Research.

[37] Chmiliar L., Mills A.J., Durepos G., Wiebe E. (2010). Multiple-Case Designs. Encyclopedia of Case Study Research.

[38] Teddlie C., Yu F. (2007). Mixed methods sampling: A typology journal of mixed methods research. J. Mix. Methods Res..

[39] Collins K.M.T., Tashakkori A., Teddlie C. (2015). Advanced Sampling Designs in Mixed Research: Current Practices and Emerging Trends in the Social and Behavioral Sciences. SAGE Handbook of Mixed Methods in Social & Behavioral Research.

[40] Faul F., Erdfelder E., Buchner A., Lang A.-G. (2009). Statistical power analyses using G*Power 3.1: Tests for correlation and regression analyses. Behav. Res. Methods.

[41] Abdi H., Williams L.J. (2010). Principal component analysis. Wiley Interdiscip. Rev. Comput. Stat..

[42] Qualtrics2020. https://www.qualtrics.com.

[43] Doosje B., Ellemers N., Spears R. (1995). Perceived Intragroup Variability as a Function of Group Status and Identification. J. Exp. Soc. Psychol..

[44] Chen G., Gully S.M., Eden D. (2001). Validation of a New General Self-Efficacy Scale. Organ. Res. Methods.

[45] The World Health Organization Quality of Life Group (1998). The World Health Organization quality of life assessment (WHOQOL): Development and general psychometric properties. Soc. Sci. Med..

[46] World Health Organization (2012). WHOQOL Manual. https://www.who.int/tools/whoqol.

[47] CDC (2018). HRQOL Methods and Measures. https://www.cdc.gov/hrqol/methods.htm.

[48] CDC (2018). Healthy Days Core Module (CDC HRQOL-4). https://www.cdc.gov/hrqol/hrqol14_measure.htm.

[49] R Core Team (2021). A Language and Environment for Statistical Computing. https://www.r-project.org/.

[50] Rosseel Y. (2012). lavaan: AnRPackage for Structural Equation Modeling. J. Stat. Softw..

[51] Zoom Video Communications, Inc. (2022). Zoom. https://zoom.us/.

[52] Turner D.W. (2010). Qualitative Interview Design: A Practical Guide for Novice Investigators. Qual. Rep..

[53] Hsieh H.-F., Shannon S.E. (2005). Three Approaches to Qualitative Content Analysis. Qual. Health Res..

[54] Paterson B., Mills A., Durepos G., Wiebe E. (2012). Within-case analysis. Encyclopedia of Case Study Research.

[55] Burns J.M.C., Mills A., Durepos G., Wiebe E. (2012). Cross-case synthesis and analysis. Encyclopedia of Case Study Research.

[56] Creswell J.W., Poth C.N. (2018). Qualitiative Inquiry & Research Design: Choosing among Five Approaches.

[57] Creswell J.W., Creswell J.D. (2018). Research Design: Qualitative, Quantitative, and Mixed Methods Approaches.

[58] Häusser J.A., Junker N.M., van Dick R. (2020). The *how* and the *when* of the social cure: A conceptual model of group- and individual-level mechanisms linking social identity to health and well-being. Eur. J. Soc. Psychol..

[59] Steffens N.K., La Rue C.J., Haslam C., Walter Z.C., Cruwys T., Munt K.A., Haslam S.A., Jetten J., Tarrant M. (2021). Social identification-building interventions to improve health: A systematic review and meta-analysis. Health Psychol. Rev..

[60] Muldoon O.T., Haslam S.A., Haslam C., Cruwys T., Kearns M., Jetten J. (2019). The social psychology of responses to trauma: Social identity pathways associated with divergent traumatic responses. Eur. Rev. Soc. Psychol..

[61] Cheng J., Diamond M. (2005). SCUBA Diving for Individuals with Disabilities. Am. J. Phys. Med. Rehabil..

[62] Walker P.A., Kampman H. (2021). “It didn’t bring back the old me but helped me on the path to the new me”: Exploring posttraumatic growth in British veterans with PTSD. Disabil. Rehabil..

[63] Henrykowska G., Soin J., Pleskacz K., Siermontowski P. (2022). Influence of Scuba Diving on the Quality of Life of People with Physical Disabilities. Healthcare.

[64] Cameron J.E., Voth J., Jaglal S.B., Guilcher S.J.T., Hawker G., Salbach N.M. (2018). “In this together”: Social identification predicts health outcomes (via self-efficacy) in a chronic disease self-management program. Soc. Sci. Med..

[65] Benyon K., Hill S., Zadurian N., Mallen C. (2010). Coping strategies and self-efficacy as predictors of outcome in osteoarthritis: A systematic review. Musculoskelet. Care.

[66] Jones F., Riazi A. (2011). Self-efficacy and self-management after stroke: A systematic review. Disabil. Rehabil..

[67] Mortenson W., Noreau L., Miller W. (2010). The relationship between and predictors of quality of life after spinal cord injury at 3 and 15 months after discharge. Spinal Cord.

[68] Wilski M., Tasiemski T. (2016). Illness perception, treatment beliefs, self-esteem, and self-efficacy as correlates of self-management in multiple sclerosis. Acta Neurol. Scand..

[69] Van Diemen T., Crul T., van Nes I., Geertzen J.H., Post M.W. (2017). Associations Between Self-Efficacy and Secondary Health Conditions in People Living With Spinal Cord Injury: A Systematic Review and Meta-Analysis. Arch. Phys. Med. Rehabil..

[70] Martinez-Calderon J., Meeus M., Struyf F., Luque-Suarez A. (2018). The role of self-efficacy in pain intensity, function, psychological factors, health behaviors, and quality of life in people with rheumatoid arthritis: A systematic review. Physiother. Theory Pract..

